# Birt-Hogg-Dubé syndrome with c.1579_1580insA variant in a Chinese family: a case report

**DOI:** 10.3389/fmed.2023.1184854

**Published:** 2023-05-03

**Authors:** Shijie Tang, Chuanqi Wei, Xiaoyu Wang, Min Xiao, Fengming Luo, Lei Chen

**Affiliations:** ^1^Department of Pulmonary and Critical Care Medicine, West China Hospital, Sichuan University, Chengdu, Sichuan, China; ^2^Department of Otolaryngology-Head and Neck Surgery, West China Hospital, Sichuan University, Chengdu, Sichuan, China

**Keywords:** Birt-Hogg-Dubé syndrome, case study, exome sequencing, genetic disease, spontaneous pneumothorax

## Abstract

Birt-Hogg-Dubé (BHD) syndrome, is a rare genetic disease with heterogeneous manifestations in different populations. In this study, we reported a Chinese female BHD case and her family members with c.1579_1580insA variant in *FLCN* gene, who were characterized by diffused pulmonary cysts/bulla, and reviewed another five familial BHD cases in China. Based on these cases, recurrent spontaneous pneumothorax is likely to be the first symptom for BHD in Chinese patients, with particularly but not limited to c.1579_1580insA variant. Therefore, attention to the early diagnosis of BHD in China should focus on pulmonary signs, but skin or kidney lesions still can not be neglected.

## Introduction

Birt-Hogg-Dubé (BHD) syndrome is a rare autosomal dominant genetic disease, which causes a clinical syndrome including benign skin tumors (fibrofolliculomas), renal tumors and pulmonary cysts/bulla via pathogenic variants at position 17p11.2 on chromosome, known as *FLCN* gene (1, 2). *FLCN* gene contains 14 exons and encodes a follicular protein consisting of 579 amino acids, which is mostly interfered through occurring code shifting or nonsense variants in *FLCN* gene, especially in organs including skin, kidney and lung (3, 4). In China, c.1285dup/delC in exon 11 of *FLCN* gene has been documented to be the most frequent variant in BHD (5, 6). Here, we reported a Chinese female BHD case and her family members with c.1579_1580insA variant in *FLCN* gene, who were characterized by diffused pulmonary cysts/bulla as well as spontaneous pneumothorax.

## Case presentation

**FIGURE 1:**
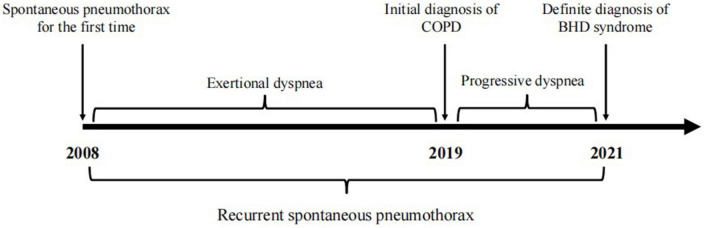
Timeline of the patient’s history of present illness.

A 65-year-old woman was admitted to the West China Hospital (WCH) of Sichuan University because of a 13-year history of dyspnea and recurrent pneumothorax. In the past 13 years before admission, the patient experienced exertional dyspnea with unknown cause and suffered from spontaneous tension pneumothorax in both sides for several times, followed by the closed drainage of chest. Two years ago, she was diagnosed in the local hospital with chronic obstructive pulmonary disease (COPD) and pulmonary bulla, and treated by budesonide formoterol powder inhaler but with poor outcomes. One day prior to admission, the symptom of dyspnea was suddenly worsened with a right-sided tension pneumothorax again. Therefore she was emergently admitted to the WCH for further treatment. The timeline of history of present illness was showcased in Figure 1. The patient has no history of smoking or other exposure. Her father, sister, brother and daughter all suffered from lung lesions (cysts or bulla).

**FIGURE 2:**
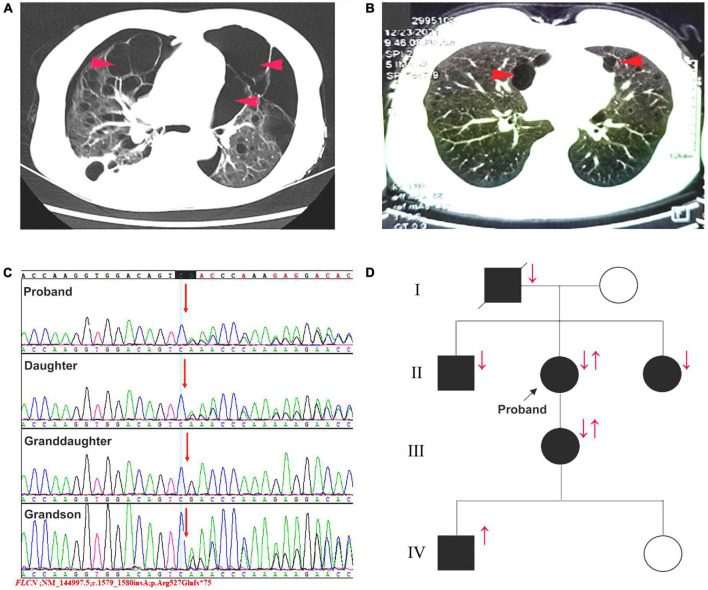
**(A)** The patient and **(B)** her daughter’s representative chest CT images. red arrows: pulmonary bulla. **(C)** Sequence map for c.1579_1580insA in exon 14 of *FLCN* gene. **(D)** The pedigree of the patient and her family members. Squares: male members; circles: female members; ↓: pulmonary lesions; ↑: c.1579_1580insA variant.

On admission, body temperature: 36.8°C; heart rate: 108 times per minute; respiratory rate: 24 times per minute; blood pressure: 162/99 mmHg. Physical examination indicated a barrel-shaped chest, hyperresonance when percussing, a few moist crackles in both lungs. No abnormality was found in skin. In the laboratory test, percentage of neutrophil in blood was a little bit higher (77%). Liver and kidney functions were normal. α1 antitrypsin and autoimmune antibodies were negative. Chest CT scan revealed pneumothorax in the right thoracic cavity, emphysema with multiple pulmonary bulla and scattered inflammatory shadows in both lungs. The representative chest CT images of the patient (A) and her daughter (B) were shown in Figure 2. Abdominal CT did not show any significant abnormality. The patient was then treated with cephalosporin and closed drainage of the right chest. After 2-week treatment, the symptoms were significantly improved and the drainage tube was removed before discharge. During the 3-month follow-up, the patient stayed at home and felt not well because of progressive dyspnea, especially after movement, and unexpectedly suffered from another pneumothorax episode. This case report was approved by the Institutional Review Board of West China Hospital of Sichuan University. The informed consent was obtained from the patient.

In this case, combined the clinical manifestations with the pulmonary bullae-related family history, we considered there might be a family hereditary disease. Consequently, the whole exome sequencing showed an insertional variant (c.1579_1580insA) in 14 exon in *FLCN* gene (Figure 2C). The patient was eventually diagnosed with BHD syndrome and the pedigree of the family was shown in Figure 2D. In addition to the present patient and her family F6 in Table 1, there were another five familial BHD cases with c.1579_1580insA variant reported in China F1–F5 in Table 1. Based on these six familial BHD cases, pulmonary cysts and pneumothorax seemed to be much more common than injury in skin and kidney.

**TABLE 1:** Clinical data of BHD patients (c.1579_1580insA) and their families in China.

Family ID	Age	Sex	Pneumo -thorax	Lung cysts	Kidney tumors	Skin lesion	c.1579_1580insA variant
F1 F1 F1	38	F	Yes	Yes	No	Yes	Yes
	–	F	Yes	Yes	–	–	–
	–	M	Yes	Yes	–	–	–
F2 F2 F2 F2 F2 F2 F2	77	F	No	Yes	–	–	Yes
	57	F	No	Yes	No	–	Yes
	55	F	Yes	–	No	–	Yes
	50	F	Yes	–	No	–	Yes
	48	F	Yes	Yes	No	–	Yes
	45	M	No	Yes	No	–	Yes
	41	F	No	Yes	No	–	Yes
F3 F3 F3 F3 F3	–	M	Yes	Yes	Yes	Yes	Yes
	–	M	Yes	Yes	Yes	No	Yes
	–	F	Yes	Yes	Yes	No	Yes
	–	F	Yes	Yes	Yes	No	Yes
	–	M	No	No	Yes	Yes	Yes
F4 F4 F4 F4 F4 F4	53	F	Yes	Yes	No	Yes	Yes
	48	F	Yes	Yes	Yes	No	Yes
	47	F	Yes	Yes	No	No	Yes
	28	M	Yes	No	No	Yes	Yes
	21	M	Yes	Yes	No	No	Yes
	18	F	No	No	No	No	Yes
F5 F5 F5 F5 F5 F5 F5 F5	–	F	Yes	Yes	No	No	Yes
	–	F	–	–	–	–	Yes
	–	F	–	–	–	–	Yes
	–	F	Yes	–	–	–	–
	–	F	Yes	Yes	–	–	–
	–	F	Yes	Yes	–	–	–
	–	M	Yes	Yes	–	–	Yes
	–	M	Yes	Yes	–	–	–
F6 F6 F6 F6 F6 F6	65	F	Yes	Yes	No	No	Yes
	–	F	No	Yes	No	No	–
	–	M	No	Yes	No	No	–
	–	M	No	Yes	No	No	–
	39	F	No	Yes	No	No	Yes
	–	M	No	–	No	No	Yes

F, female; M, male; –, not reported.

## Discussion

In the present study, after analyses of BHD cases in six families, we established a “bridge” between c.1579_1580insA variant and typical clinical feature (pulmonary signs) in Chinese BHD patients, although no significant association of specific genotype with clinical phenotype in BHD patients has been reported previously.

It was well-documented that Chinese BHD patients, compared to Caucasians, tended to have multiple pulmonary cysts, and similar to this case, spontaneous pneumothorax was often their first and even only symptom, and more than 95% of reported Chinese patients had pulmonary lesions, which was significantly higher than Caucasians (5, 7), which usually resulted in a misdiagnosis of COPD initially in China. Most of the pulmonary cysts were 0.5–6 cm in diameter, with multiple thin-walled pulmonary cysts that were lobulated and multi-segmented, and mainly located near the lower lobe of the lungs and mediastinum bilaterally (8, 9).

The pathogenic mechanisms of pulmonary cysts in BHD remain not fully understood. Previous studies indicated that BHD pulmonary cysts might be caused by dysregulations of not only epithelial-stromal interactions through *FLCN*-dependent mTOR signaling, leading to the formation of cystic alveoli (10, 11), but also cell-cell adhesions via *FLCN* interacting with P0071, a member of armadillo protein subfamily, which functioned in cell-cell adhesion by aggregating and stabilizing cadherins (12–14). Moreover, *FLCN* deficiency decreased the secretion of pulmonary surfactant through increasing permeability of alveolar epithelial cells and inducing apoptosis, leading to pathogenic changes of alveolar surface tension and decreased dynamic compliance, which indicated an increased resistance to mechanical stress (even respiratory movement), thus resulting in a potential for expansion of the cyst wall and rupture of the weak surface (15–17). Similarly, in BHD patients, rupture of the cyst wall initiated cyst formation and secondary pneumothorax, which were mainly distributed in the parts of greatest tensile force in lungs (18–20).

## Conclusion

Overall, since recurrent spontaneous pneumothorax is likely to be the first clinical presentation for BHD in Chinese patients, with particularly but not limited to c.1579_1580insA variant, attention to the early diagnosis of BHD in China should focus on pulmonary signs. Hence, it is crucial for early differentiation of pulmonary cysts, especially in younger patients, caused by BHD from other diseases, such as lymphangioleiomyomatosis (LAM), lymphocytic interstitial pneumonia (LIP), COPD, cystic fibrosis, etc. Meanwhile, inquiry of patients’ family history regarding lung lesion is also very important.

## Data availability statement

The original contributions presented in this study are included in the article/supplementary material, further inquiries can be directed to the corresponding authors.

## Ethics statement

This study was reviewed and approved by the Institutional Review Board of West China Hospital of Sichuan University. The patients/participants provided their written informed consent to participate in this study. Written informed consent was obtained from the individual(s) for the publication of any potentially identifiable images or data included in this article.

## Author contributions

ST, CW, and XW: case report, literature review, and manuscript drafting. MX: data analyses. LC and FL: conception and draft revision. All authors contributed to the article and approved the submitted version.

## References

[1] Nickerson M Warren M Toro J Matrosova V Glenn G Turner M Mutations in a novel gene lead to kidney tumors, lung wall defects, and benign tumors of the hair follicle in patients with the Birt-Hogg-Dubé syndrome. *Cancer Cell.* (2002) 2:157–64. 10.1016/s1535-6108(02)00104-6 12204536

[2] Woodford M Andreou A Baba M van de Beek I Di Malta C Glykofridis I Seventh BHD international symposium: recent scientific and clinical advancement. *Oncotarget.* (2022) 13:173–81. 10.18632/oncotarget.28176 35070081 PMC8780807

[3] Warren M Torres-Cabala C Turner M Merino M Matrosova V Nickerson M Expression of Birt-Hogg-Dubé gene mRNA in normal and neoplastic human tissues. *Mod Pathol.* (2004) 17:998–1011. 10.1038/modpathol.3800152 15143337

[4] Daccord C Good J Morren M Bonny O Hohl D Lazor R. Birt-Hogg-Dubé syndrome. *Eur Respir Rev.* (2020) 29:200042. 10.1183/16000617.0042-2020 PMC9489184 32943413

[5] Hu X Zhang G Chen X Xu K. Birt-Hogg-Dubé syndrome in Chinese patients: a literature review of 120 families. *Orphanet J Rare Dis.* (2021) 16:223. 10.1186/s13023-021-01848-8 34001170 PMC8130425

[6] Zhou W Liu K Xu K Liu Y Tian X. Clinical and genetic comparison of Birt-Hogg-Dubé Syndrome (Hornstein-Knickenberg Syndrome) in Chinese: a systemic review of reported cases. *Int J Gen Med.* (2022) 15:5111–21. 10.2147/IJGM 35637701 PMC9144823

[7] Liu K Xu W Tian X Xiao M Zhao X Zhang Q Genotypic characteristics of Chinese patients with BHD syndrome and functional analysis of FLCN variants. *Orphanet J Rare Dis.* (2019) 14:223. 10.1186/s13023-019-1198-y 31615547 PMC6794894

[8] Lee J Cha Y Kim J Choi J. Birt-Hogg-Dubé syndrome: characteristic CT findings differentiating it from other diffuse cystic lung diseases. *Diagn Interv Radiol.* (2017) 23:354–9. 10.5152/dir.2017.16606 28830849 PMC5602359

[9] Xu W Xu Z Liu Y Zhan Y Sui X Feng R Characterization of CT scans of patients with Birt-Hogg-Dubé syndrome compared with those of Chinese patients with non-BHD diffuse cyst lung diseases. *Orphanet J Rare Dis.* (2020) 15:176. 10.1186/s13023-020-01448-y 32631372 PMC7336475

[10] Furuya M Tanaka R Koga S Yatabe Y Gotoda H Takagi S Pulmonary cysts of Birt-Hogg-Dubé syndrome: a clinicopathologic and immunohistochemical study of 9 families. *Am J Surg Pathol.* (2012) 36:589–600. 10.1097/PAS.0b013e3182475240 22441547

[11] Zhong M Zhao X Li J Yuan W Yan G Tong M Tumor suppressor folliculin regulates mTORC1 through primary cilia. *J Biol Chem.* (2016) 291:11689–97. 10.1074/jbc 27072130 PMC4882437

[12] Medvetz D Khabibullin D Hariharan V Ongusaha P Goncharova E Schlechter T Folliculin, the product of the Birt-Hogg-Dube tumor suppressor gene, interacts with the adherens junction protein p0071 to regulate cell-cell adhesion. *PLoS One.* (2012) 7:e47842. 10.1371/journal.pone.0047842 23139756 PMC3490959

[13] Keil R Schulz J Hatzfeld M. p0071/PKP4, a multifunctional protein coordinating cell adhesion with cytoskeletal organization. *Biol Chem.* (2013) 394:1005–17. 10.1515/hsz-2013-0114 23640939

[14] Khabibullin D Medvetz D Pinilla M Hariharan V Li C Hergrueter A Folliculin regulates cell-cell adhesion, AMPK, and mTORC1 in a cell-type-specific manner in lung-derived cells. *Physiol Rep.* (2014) 2:e12107. 10.14814/phy2.12107 25121506 PMC4246594

[15] Goncharova E Goncharov D James M Atochina-Vasserman E Stepanova V Hong S Folliculin controls lung alveolar enlargement and epithelial cell survival through E-cadherin, LKB1, and AMPK. *Cell Rep.* (2014) 7:412–23. 10.1016/j.celrep.2014.03.025 24726356 PMC4034569

[16] Chu L Luo Y Chen H Miao Q Wang L Moats R Mesenchymal folliculin is required for alveolar development: implications for cystic lung disease in Birt-Hogg-Dubé syndrome. *Thorax.* (2020) 75:486–93. 10.1136/thoraxjnl-2019-214112 32238524

[17] Min H Ma D Zou W Wu Y Ding Y Zhu C FLCN-regulated miRNAs suppressed reparative response in cells and pulmonary lesions of Birt-Hogg-Dubé syndrome. *Thorax.* (2020) 75:476–85. 10.1136/thoraxjnl-2019-213225 32184379 PMC7279199

[18] Johannesma P Houweling A van Waesberghe J van Moorselaar R Starink T Menko F The pathogenesis of pneumothorax in Birt-Hogg-Dubé syndrome: a hypothesis. *Respirology.* (2014) 19:1248–50. 10.1111/resp.12405 25302759

[19] Kennedy J Khabibullin D Henske E. Mechanisms of pulmonary cyst pathogenesis in Birt-Hogg-Dube syndrome: the stretch hypothesis. *Semin Cell Dev Biol.* (2016) 52:47–52. 10.1016/j.semcdb.2016.02.014 26877139

[20] Yang J Hu X Li J Zhang G Ge Y Wei W. Correlative analysis of lung CT findings in patients with Birt-Hogg-Dubé Syndrome and the occurrence of spontaneous pneumothorax: a preliminary study. *BMC Med Imaging.* (2022) 22:22. 10.1186/s12880-022-00743-3 35125098 PMC8819866

